# Post-Pandemic Dynamics of the Global Circulation of Human Metapneumovirus and Respiratory Syncytial Virus

**DOI:** 10.1093/infdis/jiaf086

**Published:** 2025-07-16

**Authors:** Marie-Noëlle Billard, Joanne G Wildenbeest, Ruben Kole, Barry Rodgers-Gray, John Fullarton, Louis Bont

**Affiliations:** Department of Paediatric Infectious Diseases and Immunology, Wilhelmina Children's Hospital, University Medical Centre Utrecht, Utrecht, The Netherlands; Department of Paediatric Infectious Diseases and Immunology, Wilhelmina Children's Hospital, University Medical Centre Utrecht, Utrecht, The Netherlands; Department of Paediatric Infectious Diseases and Immunology, Wilhelmina Children's Hospital, University Medical Centre Utrecht, Utrecht, The Netherlands; Violicom Medical Limited, Aldermaston, United Kingdom; Violicom Medical Limited, Aldermaston, United Kingdom; Department of Paediatric Infectious Diseases and Immunology, Wilhelmina Children's Hospital, University Medical Centre Utrecht, Utrecht, The Netherlands; ReSViNET, Zeist, The Netherlands

**Keywords:** respiratory syncytial virus, human metapneumovirus, hMPV, seasonality, transmission

## Abstract

**Background:**

Understanding the seasonality of human metapneumovirus (hMPV) and respiratory syncytial virus (RSV) is important for public health planning. It can support rationale for using another country data to model immunization strategies where seasonality data are lacking. While some studies have investigated (sub)-national seasonality drivers, this is the first to describe global seasonality for RSV and hMPV.

**Methods:**

We included 26 countries with consistent reporting and >10 detections at the peak, after searching international databases and local reports. Weekly surveillance data from January 2022 to June 2024 were included. Viral activity was defined by comparing the 4-week moving average of the positivity rate to its annual average. “Seeding” events were the first 2 consecutive weeks with a statistically significant increase in detections.

**Results:**

Most countries showed seasonal patterns of RSV and hMPV, except for some tropical countries. The RSV peak occurred systematically before the hMPV peak. On a Mercator projection, hMPV appeared to circulate in a counterclockwise manner, opposite to RSV. Although global information was incomplete, the first seeding events occurred in a short time in multiple countries with year-to-year variations.

**Conclusions:**

We have provided critical information on the circulation of hMPV and RSV. We only found 26 countries reporting suitable surveillance data in publicly accessible reports, which likely reflects true gaps in surveillance.

Human metapneumovirus (hMPV) and respiratory syncytial virus (RSV) are common causes of lower respiratory tract infections (LRTIs), particularly in young children and older adults. In recent global systematic reviews, 643 000 (uncertainty range [UR] 425 000–977 000) LRTI hospital admissions in children <5 years of age were estimated to be associated with hMPV in 2018 while 3.6 million (UR 2.9–4.6 million) were associated with RSV in 2019 [1, 2]. In adults aged >65 years in the United States (US), the annual incidence rates were 22.1/10 000 for hMPV (95% confidence interval [CI] 12.1–33.7) and 25.4/10 000 for RSV (95% CI 13.1–38.0) [3]. Before the severe acute respiratory syndrome coronavirus 2 (SARS-CoV-2) pandemic, both hMPV and RSV were described as seasonal respiratory pathogens causing annual peaks in temperate climates [4]. While local year-to-year epidemics have been described, less is known about RSV and hMPV circulation between and within hemispheres.

As for local transmission, the global circulation dynamics of each respiratory virus are likely determined by a unique mix of factors including viral characteristics, meteorological factors, and human behavior. RSV can survive longer on surfaces and thus is more likely to be transmitted through infected surfaces than hMPV, which requires more direct contact with an infected individual [5]. Additionally, for a highly contagious pathogen, a single infected traveler can result in multiple importations at a destination following cross-infection while traveling, increasing the chances of seeding [6]. One modeling study of RSV in the US suggested that the number of importations could be one of multiple drivers of the out-of-season resurgence of RSV during the SARS-CoV-2 pandemic [7]. Meteorological factors (humidity, temperature, precipitation) can impact host susceptibility through modulation of the immune system and compromise of the mucosal barrier but also how long viruses can survive and stay in suspension [8]. The likelihood of transmission to one geographic region or another is also likely impacted by human behavior. For RSV, genetic analyses of different strains suggested that RSV may travel by airplane between countries [9]. Adding to the complexity, the drivers can vary geographically and between seasons due to confounding factors including varying contact rates, interference between viruses, use of nonpharmaceutical interventions, exceptional meteorological variations, and super-spreading events.

Understanding the dynamics of the seasonality of respiratory pathogens is important when preparing for local outbreaks [10]. Furthermore, it will help in predicting seasonality in countries with limited surveillance and irregular reporting of cases. Hospital bed capacity needs to be increased during annual epidemics of respiratory pathogens to absorb surges in hospitalizations. Anticipating the timing of the peak is important to plan public health information campaigns around preventive measures. Seasonal immunization campaigns need to be planned so that the protection period includes the expected annual peak of viral infection. In recent years, preventive products have been licensed to protect infants and older adults against RSV disease. While there is currently no vaccine licensed against hMPV, several products are at various phases of clinical development, including bivalent RSV-hMPV vaccines for older adults [11, 12]. Understanding seasonality drivers would permit the monitoring and prediction of the emergence and spread of new and possibly resistant strains.

Previous research on global circulation of RSV and hMPV has focused on comparing patterns of seasonality between regions or was limited to general north/south and east/west gradient in the timing of the season [4]. This study is a secondary analysis of publicly accessible surveillance data for RSV and hMPV during 2 consecutive seasons. Its objective was to identify the main routes of infection transfer within and between hemispheres. To our knowledge, this article is the first to analyze the dynamics of the circulation of RSV and hMPV within and between hemispheres.

## METHODS

### Design

This study was a retrospective analysis of publicly accessible surveillance data of RSV and hMPV from January 2022 to June 2024. The primary objective was to describe and characterize the main routes of circulation between and within hemispheres during 2 consecutive seasons.

### Country Selection

**Figure 1.**
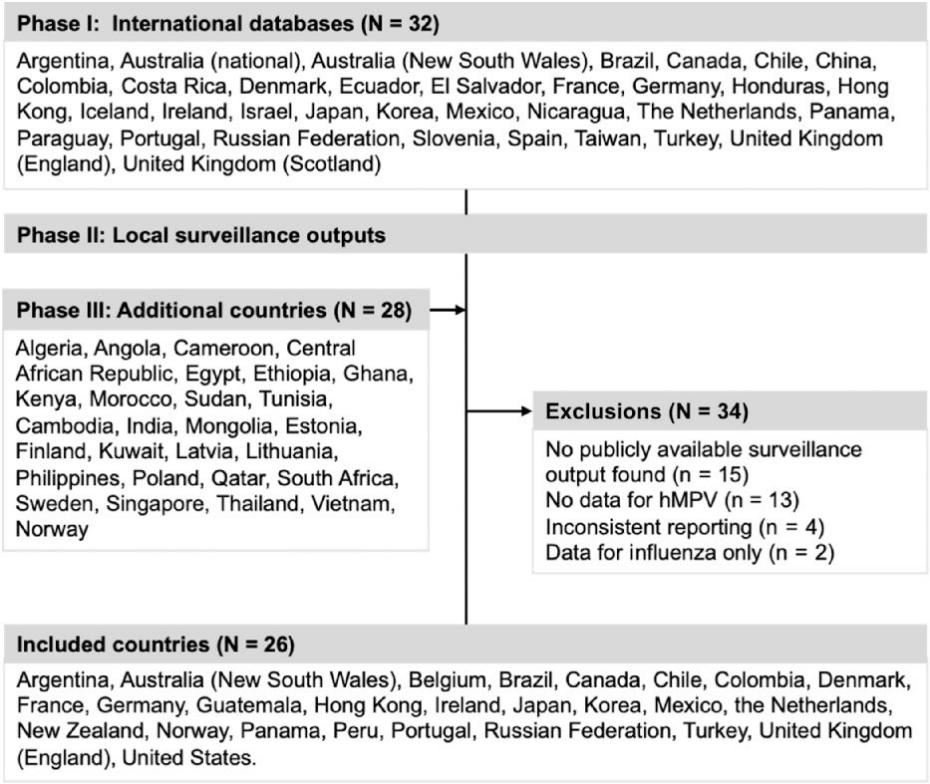
Flowchart of the inclusion of countries. Abbreviation: hMPV, human metapneumovirus.

The search for eligible countries was conducted in 3 phases (Figure 1). First, large international databases were used to identify countries most likely to report RSV and hMPV surveillance data (FluNet database, World Health Organization Global Influenza Surveillance and Response System; Atlas of Infectious Diseases, European Centre for Disease Prevention and Control). Second, local surveillance outputs were reviewed to assess the regular availability of the required data. Countries with at least 10 detections reported at the peak and <4 missing weeks in 2022 at the time of the search (October–December 2022) were considered eligible. Information from these countries’ existing surveillance systems was also extracted. Third, local surveillance outputs from arbitrarily selected additional countries were included to increase geographic diversity. No other inclusion criteria were used, since a high level of heterogeneity between countries and surveillance systems had been anticipated. In total, 26 countries with suitable data were included; 18 were high-income countries and 8 were middle-income countries (Supplementary Table 1). According to the latitude of the capital city, 7 were classified as tropical countries (25°N to 25°S) and 19 were classified as temperate countries, including 4 from the Southern Hemisphere (>25°S) and 14 from the Northern Hemisphere (>25°N).

### Data Sources

All data sources used are listed in Supplementary Table 1. Overall, 10 data sources relied on reporting from laboratories with no indication of the origin of the samples or the case definition used. Other sources were sentinel surveillance in hospital (n = 7), in primary care (n = 5), or mixed (n = 4). Case definitions varied between countries. Hospital-based surveillance might have included outpatient clinics. In 20 of 26 countries, the numbers of RSV and hMPV detections were reported among the same number of samples tested, suggesting that all (or the same subset of) samples were tested for both viruses. In the remaining 6 countries, the number of tests performed was substantially lower for hMPV than for RSV. In France, Denmark, the Russian Federation, and Norway, surveillance was functioning only during the winter. We assumed no detection and 0% positivity rate in the rest of the year. Other countries had year-round surveillance. When reported, the surveillance was representative of the entire country (n = 14/15).

### Data Extraction

For each country and virus, we extracted the weekly number of detections, the weekly number of tests performed, and the weekly positivity rate. The positivity rate was defined as the number of positive results per test performed. When the number of positive cases was reported in the absence of the number of tests, the total number of samples tested was estimated from the total number of detections for any respiratory virus (this applied to data from the Netherlands).

### Analyses

The annual RSV and hMPV seasons in each country were described with the “seeding” date, periods of increased viral activity, and the peak. The infection “seeding” date was defined as the last day of the first week of 2 consecutive weeks with a statistically significant increase in the number of detections during the period immediately before a period of increased viral activity as defined below. An increase in the number of detections was identified using a statistically significant binomial test (*P* < .05) comparing the number of detections among the total number of tests performed in 1 week to the previous week. Due to differences in the type and scale of surveillance systems for RSV and hMPV, the annual number of tests done ranged from 500–1000 to >1 000 000 and the annual number of detections ranged from <50 to >10 000. To enable comparisons between countries, viral activity levels for each country and virus were calculated solely from each country's own data. A baseline positivity rate was calculated as the average positivity rate over 2 complete annual cycles to account for seasonal variation (104 weeks from week 27 of 2022 to week 26 of 2024). Viral activity was defined by comparing the 1-week or the 4-week average positivity rate to the country's own baseline rate and its standard deviation. Four categories were defined: no/low level (positivity rate < baseline); moderate level (positivity rate > baseline), high level (positivity rate > baseline + 1 × standard deviation); very high level (positivity rate > baseline + 2 × standard deviations). The period of increased viral activity was defined as consecutive weeks with viral activity from moderate to very high, with a 2-week gap tolerance. Supplementary Figure 3 shows the weekly number of detections, the weekly positivity rate, and the viral activity levels for RSV and hMPV in a selection of countries. The peak was defined as the week with the highest number of detections reported during a period of increased viral activity. We did not define peaks for periods of viral activity that included the first or the last week of the observation period, as more detections could have been observed outside of the observation period.

Analyses were performed using R (version 4.1.3) and Excel (for Microsoft 365) software.

## RESULTS

### Patterns of Seasonality

**Figure 2.**
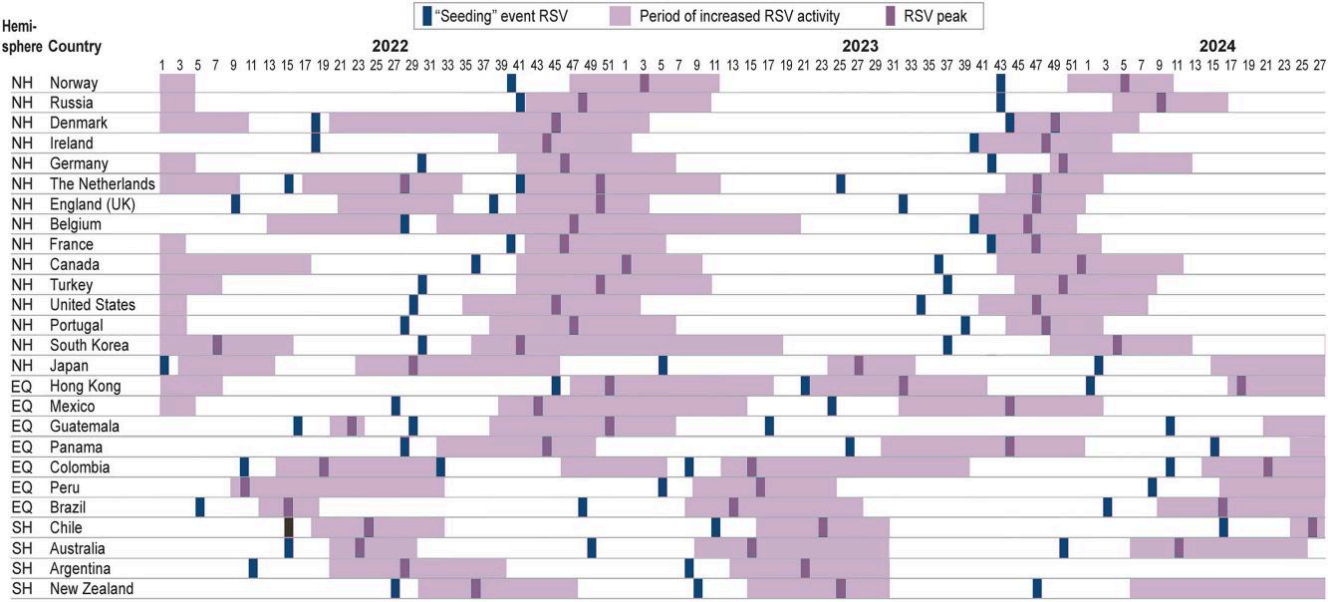
Periods of increased positivity rates and timing of the peak and “seeding” event of respiratory syncytial virus (RSV) from January 2022 to June 2024, in equatorial countries (EQ) and countries from the Northern Hemisphere (NH) and Southern Hemisphere (SH) included in the study.

**Figure 3.**
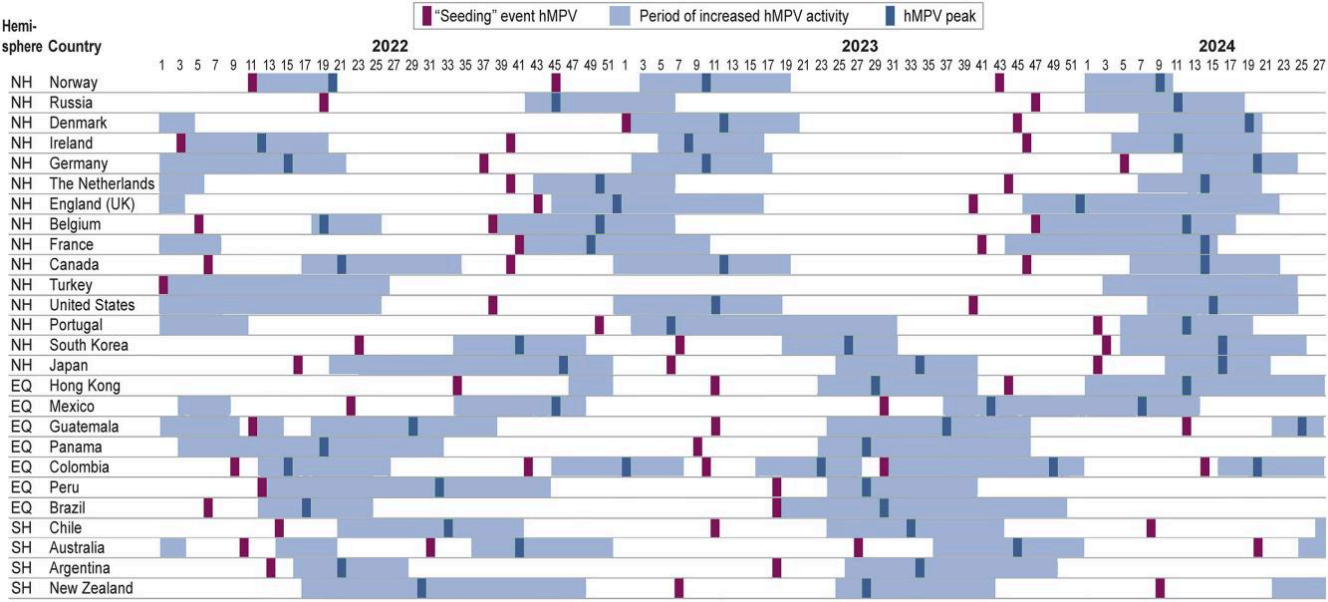
Periods of increased positivity rates and timing of the peak and “seeding” event of human metapneumovirus (hMPV) from January 2022 to June 2024, in equatorial countries (EQ) and countries from the Northern Hemisphere (NH) and Southern Hemisphere (SH) included in the study.

Overall, RSV and hMPV displayed seasonality in almost all countries, with periods of high and low viral activity (Figure 2 and Figure 3). In temperate countries, increased RSV activity was generally observed in the months of late autumn and winter—between September and February (week 35 to week 10) in Northern Hemisphere countries and between April and July (week 13 to week 30) in Southern Hemisphere countries. RSV seasonality was not clear in 3 tropical countries (Colombia, Guatemala, and Hong Kong). Other tropical countries from the Northern Hemisphere (Mexico) or Southern Hemisphere (Brazil, Peru, and Panama) exhibited similar patterns to those of temperate countries. In temperate countries, hMPV peaks tended to be later in winter and spring than the RSV peak: between November and May (week 45 to week 20) in Northern Hemisphere countries and between June and October (week 24 to week 44) in Southern Hemisphere countries. No tropical countries other than Brazil showed clear hMPV seasonality.

The circulation patterns of hMPV and RSV were not concomitant; the hMPV peak occurred systematically after the RSV peak in temperate countries from both hemispheres but not in all tropical countries (Figure 2 and Figure 3). Yet, there was some overlap between the periods of increased positivity rates (viral activity moderate to very high) for the 2 viruses. There was substantial variation between countries and seasons in how long increased activity was observed for at least 1 of the 2 viruses (from 3 months in Norway in 2023–2024 to 10 months in Portugal in 2022–2023). Part of the variation could be attributable to residual disruption of RSV and hMPV seasonality after the SARS-CoV-2 pandemic. In 2022, clear signs of residual disruption, including summer circulation, were observed in the Netherlands, England, South Korea, Japan, and Hong Kong. Seasonality had visually returned to prepandemic patterns in 2023.

In countries that displayed seasonality, the periods of increased activity for hMPV and RSV were observed approximately at the same period each year between 2022 and 2024 (Figure 2 and Figure 3). While the peaks of hMPV were observed within 10 weeks of each other year-on-year, RSV peaks were more consistent, occurring within 5 weeks of each other. Interestingly, in South Korea, where there were clear signs of disrupted seasonality with 2 RSV peaks observed in 2022, a peak of hMPV was consistently observed after the peak of RSV. The timing of the peak was difficult to determine in in Belgium, New Zealand, and Turkey due to small numbers of detections.

**Figure 4.**
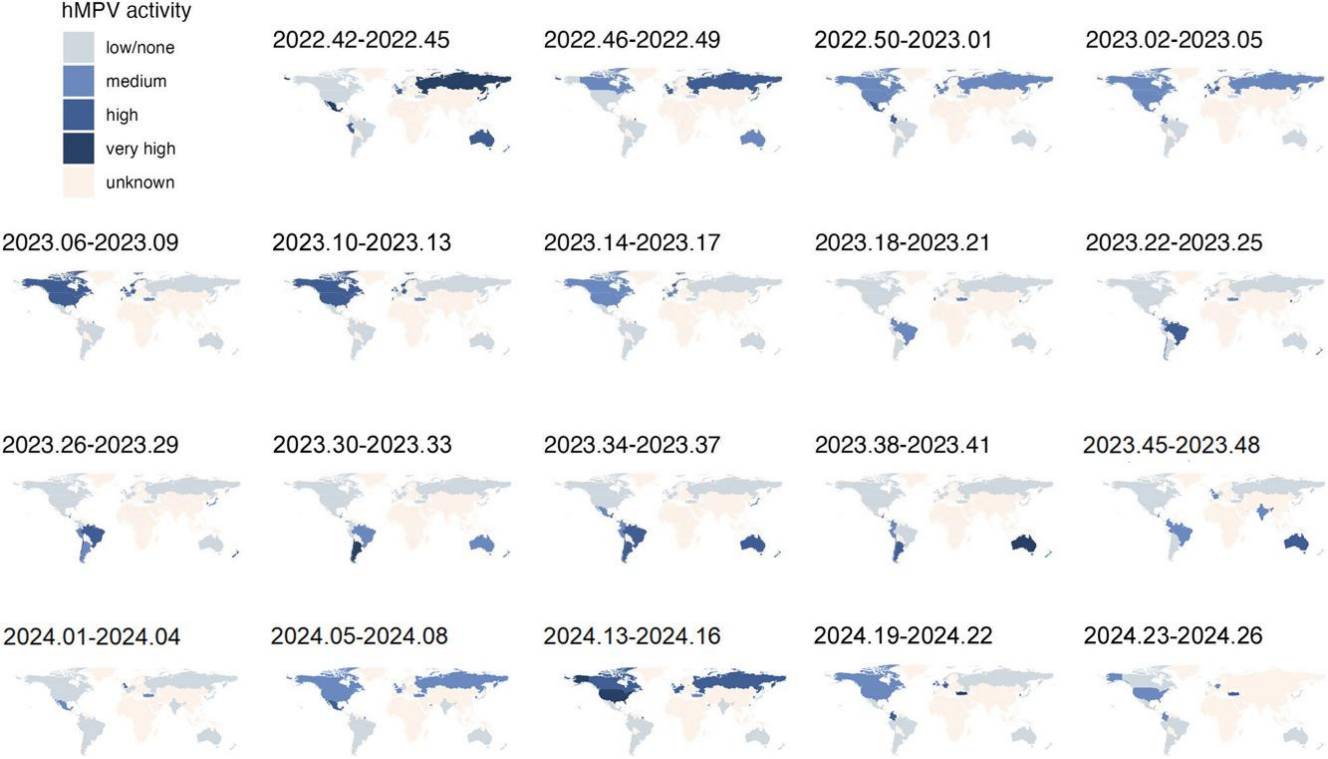
Global circulation of human metapneumovirus (hMPV), -week average, October 2022 through June 2024.

**Figure 5.**
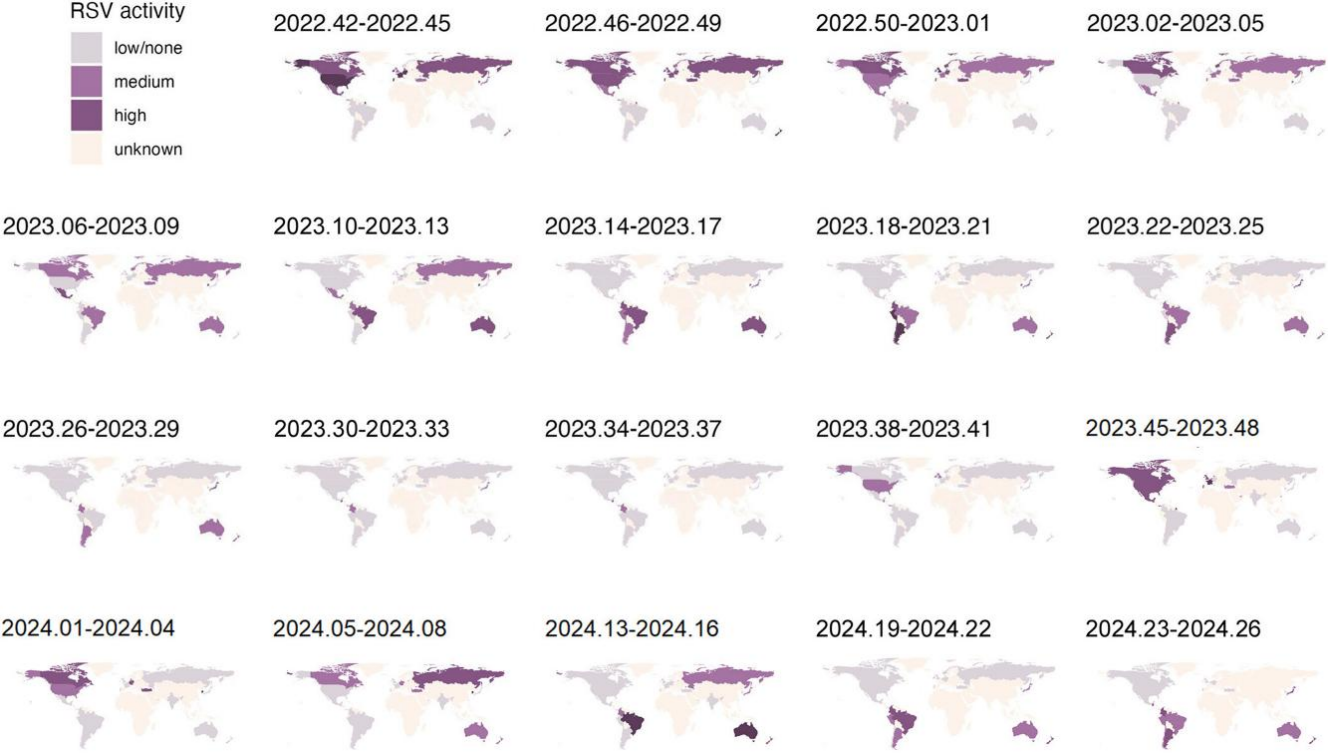
Global circulation of respiratory syncytial virus (RSV), -week average, October 2022 through June 2024.

To observe the global circulation dynamics of hMPV and RSV, the viral activity between October 2022 and June 2024 was plotted on a Mercator projection (Figure 4, Figure 5, and Supplementary Figures 1 and 2). Observation of the global dynamics, on this projection, revealed an apparent counterclockwise circulation for hMPV while RSV circulated in a clockwise manner from October 2022 to June 2024.

### Potential Routes of Transmission

For hMPV in the Southern Hemisphere, the first seeding events were detected in Central America and New Zealand (Figure 3). In Panama, a “seeding” event was observed in March 2023 (week 10). In Guatemala, “seeding” events were detected in March 2022 (week 10) and March 2023 (week 12). While in 2022 the “seeding” event was detected early in Brazil (February, week 6), it occurred later in the season in 2023 (May, week 18). In New Zealand, seeding events were detected in February 2023 (week 7) and March 2024 (week 9).

In the Northern Hemisphere, the first seeding events for hMPV during the 2022–2023 and the 2023–2024 season were observed in Mexico in May 2022 (week 22) and July 2023 (week 30) (Figure 3). In both seasons “seeding” events were subsequently observed in the US and a mix of Western European countries. In the US, “seeding” events were observed in September 2022 (week 38) and October 2023 (week 40). In Europe during the 2022–2023 season, the first “seeding” events were observed in September 2022 in Germany (week 37) and Belgium (week 38). During the 2023–2024 season, the first “seeding” events were observed in October in the United Kingdom (UK) (week 40) and France (week 41).

For RSV, the first “seeding” events detected in temperate countries from the Southern Hemisphere, during the 2023 and 2024 season, were observed in Brazil and Australia (Figure 2). In Brazil, seeding events were observed in December 2022 (week 48) and January 2024 (week 3). In Australia, seeding events were observed in December 2022 (week 49) and December 2023 (week 50). In South and Central America, subsequent “seeding” events were observed in Peru (week 5 in 2023 and week 8 in 2024), Colombia (week 8 in 2023 and week 10 in 2024), and Argentina (week 8 in 2023).

For RSV in temperate countries in the Northern Hemisphere (Figure 2), Mexico, the UK, and the Netherlands were among the first countries in which “seeding” events were detected for the 2022–2023 and 2023–2024 seasons. In Mexico, seeding events were observed in July 2022 (week 27) and June 2023 (week 24). In the UK, seeding events were detected in September 2022 (week 37) and August 2023 (week 31). And in the Netherlands, seeding events were detected in October 2022 (week 41) and June 2023 (week 25).

## DISCUSSION

We analyzed publicly accessible surveillance data of hMPV and RSV from 26 countries in both hemispheres, aimed at describing global circulation dynamics of the 2 viruses. From 2022 to 2024, RSV and hMPV showed seasonal patterns in most countries although not in all tropical countries. Where RSV and hMPV were seasonal, RSV activity was observed earlier than hMPV activity, with some overlap between the 2 viruses. Despite incomplete global surveillance data, there was an apparent anticlockwise circulation of hMPV on a Mercator projection while RSV circulated in a clockwise manner. By estimating the “seeding” date, defined as the first evidence of an increase in the number of cases in a country, we assessed likely transmission routes of RSV and hMPV between hemispheres. For RSV and hMPV, the first “seeding” events were observed in multiple geographic regions almost concomitantly and in different countries year-on-year.

We observed an overlap between periods of increased activity for RSV and hMPV activity in most countries included. A recent systematic review reported no co-circulation of RSV and hMPV and hypothesized the existence of negative viral interference between RSV and hMPV [4]. This discrepancy might have resulted from different definitions of co-circulation. As in the systematic review, we did not observe concomitant peaks of RSV and hMPV. In countries with seasonal patterns, RSV peaked systematically earlier than hMPV. This was consistent with temporal correlation, possibly due to viral interference between RSV and hMPV. However, we did not fit models to detect a temporal correlation as it was outside of the scope of this article. Alternatively, interference of RSV or hMPV with other respiratory pathogens, including a negative interference with influenza [13], could have contributed to our observation. Overall, we observed large variations in the duration of the period of increased activity of at least 1 of the 2 viruses (3–10 months). This could have implications on the potential of a bivalent RSV and hMPV vaccine if the protection conferred by the vaccine was short-lived, requiring seasonal vaccination.

From 2022 to 2024, we observed an apparent anticlockwise circulation of hMPV on a Mercator projection whereas RSV appeared to circulate in a clockwise manner. The apparent difference in the sense of circulation of RSV and hMPV likely reflects differences in where transmission between the hemispheres is first observed and in which direction RSV and hMPV spread through the Americas. While hMPV appeared to travel through the Americas from north to south, RSV seemed to spread from south to north. The geographic location of the first “seeding” events was not consistent from one year to the next and generally included countries from different geographic regions like Mexico and Western Europe. This observation might suggest multiple repeated importations of cases prior to “seeding.” The number of importations was also shown to be one of the drivers of the amplitude of the summer peak of 2021 at the reemergence of RSV during the SARS-CoV-2 pandemic in the US [7]. However, it is also possible that the first season included in the analysis (2022 and 2022–2023) was not representative of a typical winter season due to residual disruption from the SARS-CoV-2 pandemic.

It is possible that RSV and hMPV are transmitted via air travel between hemispheres. As air travel allows infective agents to spread in geographically large jumps, it renders transmission routes difficult to identify in the absence of genetic sequencing data linking cases from one country to another. This hypothesis would be consistent with our observation of multiple seeding dates in various countries, sometimes not at contact point between hemispheres. A global molecular surveillance network of genetic variants of RSV recently showed that new RSV strains spread via air travel [9]. Within Europe and the Americas where the density of countries included was highest, the timing of the RSV and the hMPV season seemed consistent with land transmission between countries with common borders. In South America, seeding “events” of RSV and hMPV were observed earlier in the east than in the west, starting from Brazil, which was opposite to prevailing winds, which suggest that travelers are most likely to be the major contributors to the spread of these viruses.

Despite an extensive search for publicly accessible surveillance data, only 26 countries with sufficient data were found. Southeast Asia, Africa, and the Middle East were underrepresented or not represented at all. This limited our ability to identify the timing and location of transmission of RSV and hMPV between hemispheres. The search strategy for country was not a systematic review as surveillance data are largely unpublished. Thus, some eligible countries may have been missed. However, the uneven geographic distribution with a concentration of data in the Northern Hemisphere from high-income countries likely reflects true disparities in surveillance capacities. Despite substantial efforts to develop RSV surveillance in the last years, there is still a gap in hMPV surveillance [14]. Contrarily to RSV, there are no rapid tests available for hMPV. Thus, hMPV detections are reported when at least part of the samples received by the surveillance system are tested using multiplex polymerase chain reaction, which is costly. This gap is concerning as surveillance is an important component of understanding seasonality, preparing for vaccine implementation, and monitoring the emergence of resistant strains.

The main strength of this study is its international scale. We gathered data from 26 countries across both hemispheres over 2 consecutive seasons in the postpandemic era. This study, however, had some limitations. First, large geographic regions were underrepresented or not represented at all. Second, surveillance systems and testing practices were disrupted or adjusted during the SARS-CoV-2 pandemic. Residual disruption of seasonality was observed in multiple countries in 2022 due to the pandemic. Thus, circulation patterns were assessed from October 2022 to June 2024, excluding most data from 2022. However, it is possible that our results are partially based on atypical patterns as seasonality of other respiratory pathogens is still disrupted [15]. Third, the data were incomplete for the 2021–2022 season in the Northern Hemisphere and for the 2024 season in the Southern Hemisphere. Fourth, little information was found on the surveillance systems, testing practices, and the type of tests used. Fifth, differences between surveillance systems prevented direct comparisons between countries while differences in testing schemes for hMPV and RSV limited comparisons between the 2 viruses. In some countries, surveillance was ongoing during the winter season only. Assuming no detections during summer in countries using seasonal surveillance may have resulted in an overestimation of viral activity and delayed detection of seeding events. Also, we used the total number of detections of any respiratory virus as proxy of the total number of tests done when the total number of tests done was not known, which caused positivity rates to depend on the circulation of other respiratory viruses. However, in most surveillance systems the number of weekly samples tested can also vary according to the number of cases meeting the case definition and, by extension, according to the circulation of other pathogens causing similar symptoms. Overall, the definition of viral activity seemed sufficiently robust to detect periods of increased viral activity. However, we were not able to ascertain that the surveillance methodology was consistent throughout the entire observation period in all countries, which may have impacted the detection of periods of increased activity. Furthermore, this method might be better suited to countries with strong seasonality and >50 annual cases reported as it could otherwise result in long or multiple periods of increased viral activity.

Overall, although this analysis did not allow us to pinpoint the transmission routes of hMPV and RSV between hemispheres, it has provided more information on the circulatory patterns of both viruses around the globe. Despite an extensive search, we only found 26 countries reporting RSV and hMPV surveillance data in publicly accessible reports. While some eligible countries may have been missed, it is likely that the underrepresentation of Africa, the Middle East, and Southeast Asia reflects a true gap in surveillance. An increased level of surveillance globally will enable a more detailed understanding of the global circulation dynamics of the 2 viruses and thereby enable prevention strategies to be designed and implemented more effectively.

## Supplementary Material

jiaf086_Supplementary_Data

## Supplementary Data

Supplementary materials are available at *The Journal of Infectious Diseases* online (http://jid.oxfordjournals.org/). Supplementary materials consist of data provided by the author that are published to benefit the reader. The posted materials are not copyedited. The contents of all supplementary data are the sole responsibility of the authors. Questions or messages regarding errors should be addressed to the author.

## Notes

***Acknowledgments.*** We would like to thank the individuals and organizations responsible for producing and maintaining the surveillance data used in this study.

***Author contributions.*** M.-N. B.: Conceptualization, methodology, investigation, writing–original draft, supervision. J. G. W.: Supervision, writing–review & editing. R. K.: Data curation, writing–review & editing, investigation. B. R.-G.: Formal analysis, conceptualization, writing–original draft, writing–review & editing. J. F.: Formal analysis, writing–original draft, writing–review & editing. L. B.: Funding acquisition, conceptualization, methodology, writing–review & editing.

***Disclaimer.*** The funding body had no role in the data analysis or the preparation of the manuscript.

***Financial support.*** This study was supported by funding from AstraZeneca, Cambridge, UK.

***Supplement sponsorship.*** This article appears as part of the supplement “Human Metapneumovirus Disease Burden in Older Adults and Implications for Adult Vaccination Strategies,” sponsored by AstraZeneca.
