# Supplementary material for: Post-Pandemic Dynamics of the Global Circulation of Human Metapneumovirus and Respiratory Syncytial Virus
Source: J Infect Dis. 2025 Jul 16;232(Suppl 1):S10–8. doi: 10.1093/infdis/jiaf086 (PMC12265059; doi:10.1093/infdis/jiaf086)

## **Supplementary material**

Title: post-pandemic dynamics of the global circulation of human metapneumovirus and respiratory syncytial virus

Supplementary Figure S1: Animation of global circulation of hMPV, January 2022 through June 2024 (available at: <https://raw.githubusercontent.com/mnbil/PublicOutputs/refs/heads/main/hMPV.gif> )


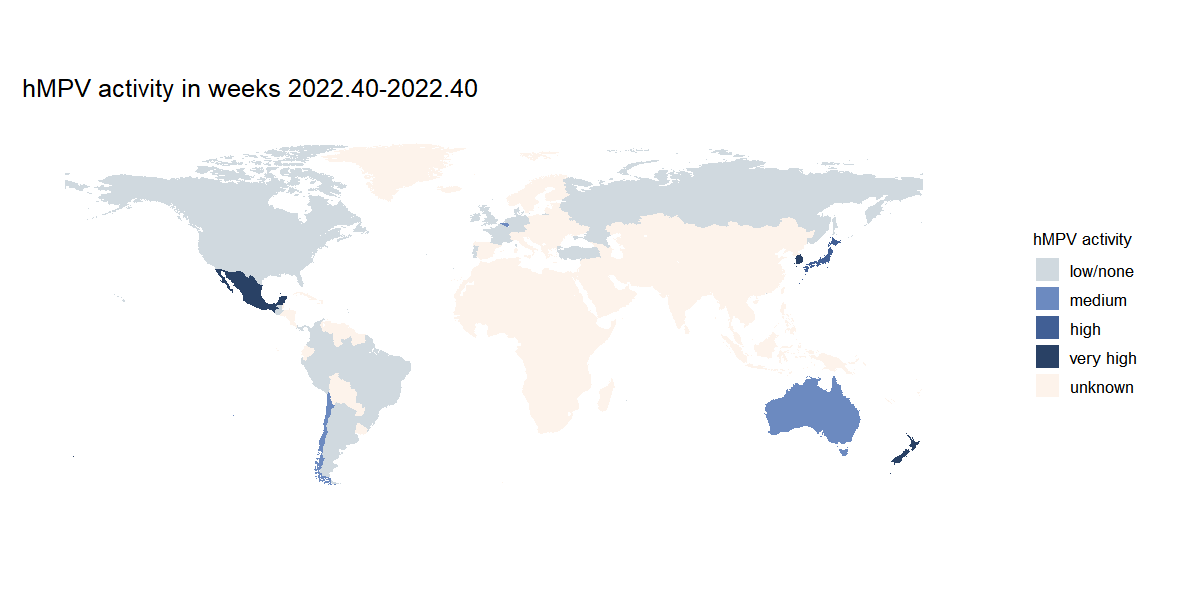


Supplementary Figure S2: Animation of global circulation of RSV, January 2022 through June 2024 (available at: <https://raw.githubusercontent.com/mnbil/PublicOutputs/refs/heads/main/RSV.gif> )


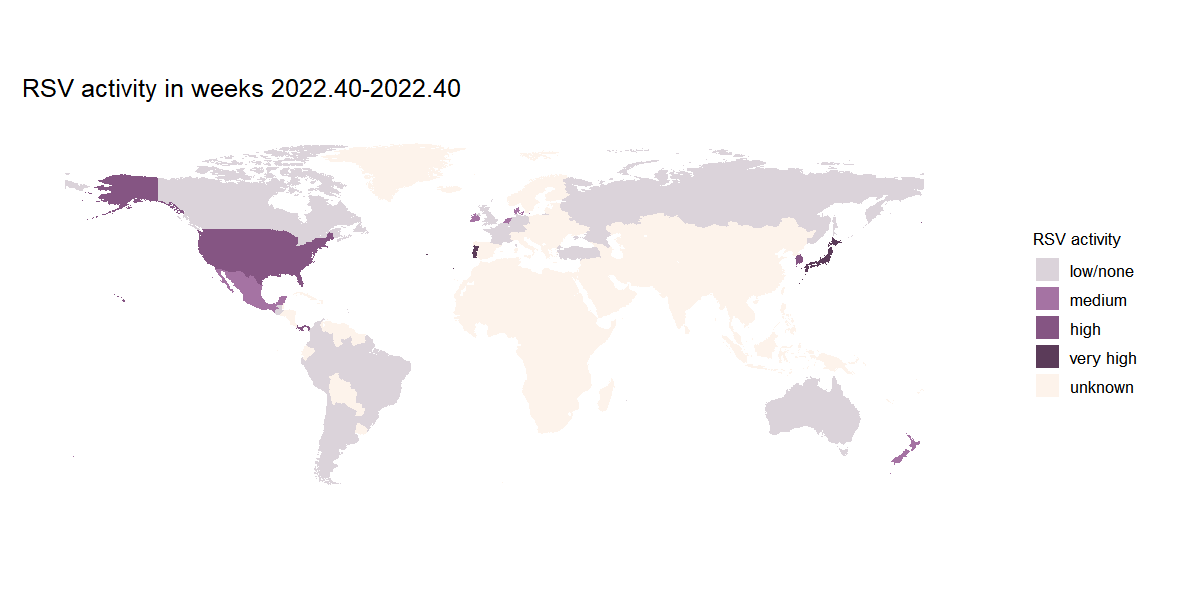


Supplementary Table 1: Data source of the 26 countries included.

| **Country** | **Geographic representativity** | **Country income classification (2024)** | **Latitude zone of capital city*** | **Data source** | **Type of surveillance** | **Samples origin** | **Case definition**** | **Annual number of RSV detections** | **Annual number of hMPV detections** | **Annual number of RSV tests** | **Annual number of hMPV tests** |
| --- | --- | --- | --- | --- | --- | --- | --- | --- | --- | --- | --- |
| **Argentina** | National | Upper-middle income | Temperate / Subtropical | FluNet (GISRS) | Non-Sentinel | Laboratories | NR | >10,000 | 5,000 – <10,000 | 100,000 – <1,000,000 | Same as for RSV |
| **Australia (NSW)** | State | High-income | Temperate / Subtropical | NSW Health | Sentinel | Laboratories | NR | >10,000 | >10,000 | >1,000,000 | Same as for RSV |
| **Belgium** | NR | High-income | Temperate / Subtropical | Sciensano | Sentinel | Primary care | ILI & ARI | 100 - <500 | 50-<100 | 500-1,000 | Same as for RSV |
| **Brazil** | NR | Upper-middle-income | Tropical / Equatorial | SIVEP-Gripe | Passive | Hospitals | SARI | >10,000 | 1,000 – <5,000 | 10,000 - <100,000 | Same as for RSV |
| **Canada** | National | High-income | Temperate / Subtropical | Health Canada | Sentinel | Laboratories | NR | >10,000 | 5,000 – <10,000 | >1,000,000 | 100,000 – <1,000,000 |
| **Chile** | NR | High-income | Temperate / Subtropical | Public Health institute | Sentinel | Hospitals | NR | >10,000 | 5,000 – <10,000 | 100,000 – <1,000,000 | Same as for RSV |
| **Colombia** | NR | Upper-middle-income | Tropical / Equatorial | FluNet (GISRS) | Non-sentinel | Laboratories | NR | 1,000 – <5,000 | 500 – <1,000 | 10,000 - <100,000 | Same as for RSV |
| **Denmark** | National | High-income | Temperate / Subtropical | Statens Serum Institut | Sentinel | Primary care | ILI | 100 - <500 | 100 - <500 | 5,000 – <10,000 | Same as for RSV |
| **England (UK)** | National | High-income | Temperate / Subtropical | Public Health England | non-sentinel | Laboratories | NR | 5,000 – <10,000 | 1,000 – <5,000 | 100,000 – <1,000,000 | 100,000 – <1,000,000 |
| **France** | National | High-income | Temperate / Subtropical | Reseau Sentinelles | Sentinel | Primary care | ARI | 100 -< 500 | 100 - <500 | 5,000 – <10,000 | Same as for RSV |
| **Germany** | National | High-income | Temperate / Subtropical | Robert Koch Institute | Sentinel | Primary care | ARI | 500 – <1,000 | 100 - <500 | 5,000 – <10,000 | Same as for RSV |
| **Guatemala** | NR | Lower-middle-income | Tropical / Equatorial | FluNet (GISRS) | Sentinel | Primary care | ILI | 100 - <500 | 100 - <500 | 1,000 – <5,000 | Same as for RSV |
| **Hong Kong** | National | High-income | Tropical / Equatorial | Center for Health protection | Sentinel | Laboratories | NR | 5,000 – <10,000 | 1,000 – <5,000 | 100,000 – <1,000,000 | Same as for RSV |
| **Ireland** | National | High-income | Temperate / Subtropical | Health Protection Surveillance Center | non-sentinel | Laboratories | NR | 100 - <500 | 100 - <500 | 5,000 – <10,000 | Same as for RSV |
| **Japan** | NR | High-income | Temperate / Subtropical | National institute of infectious diseases | Sentinel | Hospital & primary care | NR | 500 – <1,000 | 100 - <500 | 5,000 – <10,000 | Same as for RSV |
| **Mexico** | NR | Upper-middle-income | Tropical / Equatorial | FluNet (GISRS) | Sentinel | Hospital & primary care | ILI/SARI | 1,000 – <5,000 | 100 - <500 | 10,000 - <100,000 | Same as for RSV |
| **New Zealand** | National | High-income | Temperate / Subtropical | ESR | Sentinel | Hospital | SARI | 100 - <500 | 100 - <500 | 1,000 – <5,000 | 1,000 – <5,000 |
| **Norway** | National | High-income | Temperate / Subtropical | Public Health institute | non-sentinel | Hospital | NR | >10,000 | 1,000 – <5,000 | 100,000 – <1,000,000 | 100,000 – <1,000,000 |
| **Panama** | NR | High-income | Tropical / Equatorial | FluNet (GISRS) | Sentinel | Hospital & primary care | ILI/SARI | 1,000 – <5,000 | 100 - <500 | 5,000 – <10,000 | Same as for RSV |
| **Peru** | NR | Upper-middle-income | Tropical / Equatorial | FluNet (GISRS) | Sentinel | Hospital & primary care | ILI/SARI | 100 - <500 | <50 | 5,000 – <10,000 | Same as for RSV |
| **Portugal** | National | High-income | Temperate / Subtropical | National institute of health | Non-sentinel | Hospital | NR | 5,000 – <10,000 | 1,000 – <5,000 | 100,000 – <1,000,000 | Same as for RSV |
| **Russian Federation** | NR | Upper-middle-income | Temperate / Subtropical | Ministry of health | Passive | Laboratory | NR | 5,000 – <10,000 | 1,000 – <5,000 | 100,000 – <1,000,000 | Same as for RSV |
| **South Korea** | National | High-income | Temperate / Subtropical | Korea CDC | Passive | Hospitals | ARI | >10,000 | 5,000 – <10,000 | 10,000 - <100,000 | Same as for RSV |
| **The Netherlands** | National | High-income | Temperate / Subtropical | RIVM (public health institute) | Sentinel | Laboratories | NR | 1,000 – <5,000 | 1,000 – <5,000 | 10,000 - <100,000 | Same as for RSV |
| **Turkey** | NR | Upper-middle-income | Temperate / Subtropical | Ministry of health | Sentinel | Hospital | SARI | 100 - <500 | <50 | 5,000 – <10,000 | Same as for RSV |
| **United States** | National | High-income | Temperate / Subtropical | CDC (NREVSS) | Passive | Laboratory | NR | >10,000 | >10,000 | >1,000,000 | >1,000,000 |

PAHO: Pan-american health organization, NSW: new South Wales, CDC Centers for diseases control and prevention.

* Latitude zones based on the latitude of the capital city: Tropical/Equatorial (25° N to 25 ° S), Temperate / Subtropical (>25° N to >25 ° S)

**May include variations to the WHO definitions.

Supplementary Figure S3: Weekly number of RSV and hMPV tests, and of RSV and hMPV detections from January 2022 to June 2024 and visualization of the viral activity levels (mild to very high) in a selection of countries.


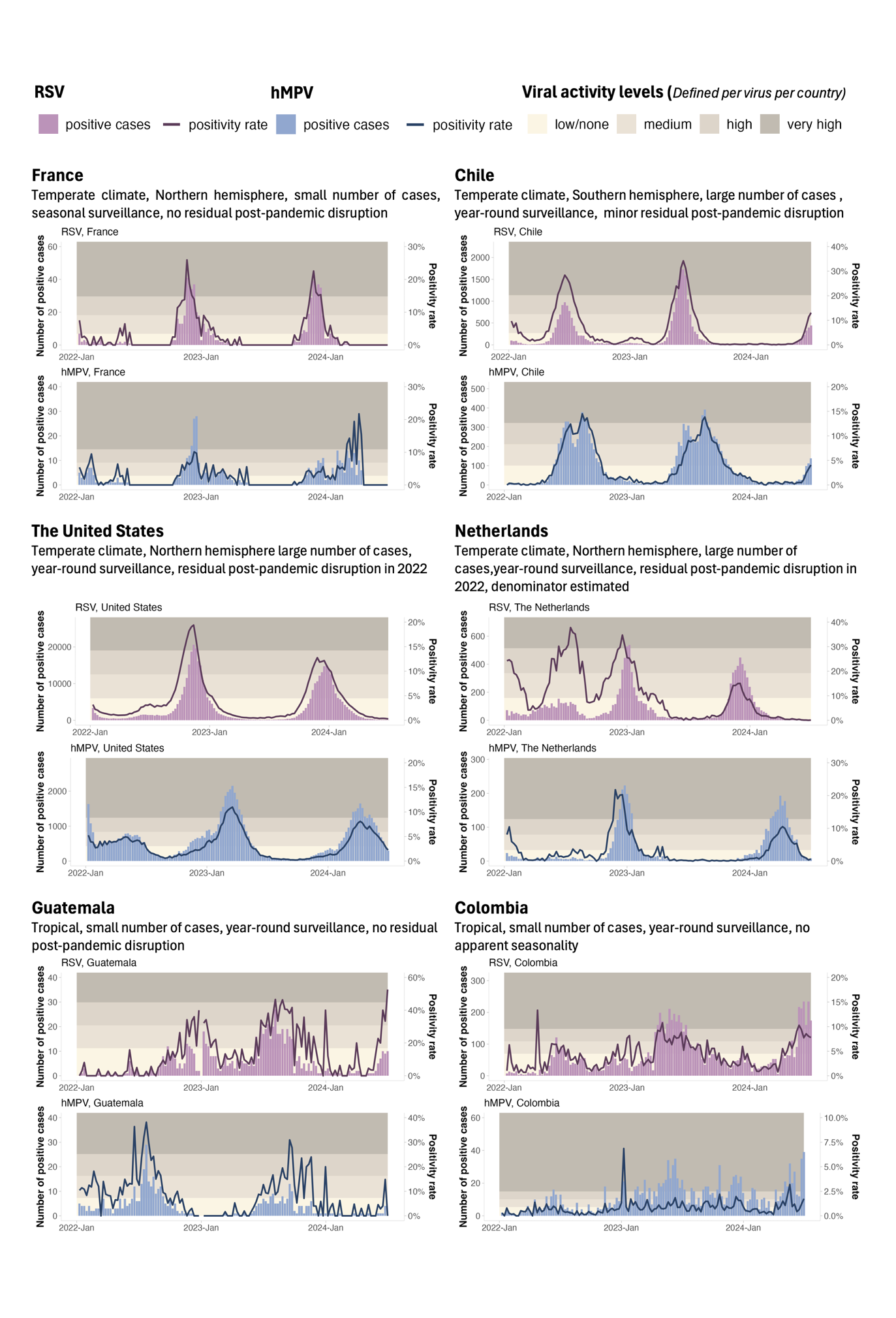

Supplement: jiaf086_Supplementary_Data [file jiaf086_supplementary_data.docx]
